# Battery longevity of a helix-fixation dual-chamber leadless pacemaker: results from the AVEIR DR i2i Study

**DOI:** 10.1093/europace/euaf074

**Published:** 2025-06-06

**Authors:** Vivek Y Reddy, Rahul Doshi, James E Ip, Pascal Defaye, Derek V Exner, Robert Canby, Morio Shoda, Maria G Bongiorni, Gerhard Hindricks, Petr Neužil, Thomas Callahan, Nima Badie, Matthew G Fishler, Reinoud E Knops

**Affiliations:** Helmsley Electrophysiology Center, Mount Sinai Fuster Heart Hospital, Icahn School of Medicine at Mount Sinai, 1190 5th Ave, New York, NY 10029, USA; Department of Clinical Cardiac Electrophysiology, HonorHealth Cardiac Arrhythmia Group, Scottsdale, AZ, USA; Department of Cardiology, Weill Cornell Medicine/New York Presbyterian Hospital, New York, NY, USA; Department of Cardiology and Vascular Medicine, CHRU Albert Michallon, Grenoble, France; Department of Cardiac Sciences, Foothills Medical Centre, Calgary, Canada; Department of Cardiology, Texas Cardiac Arrhythmia Institute, Austin, TX, USA; Department of Cardiology, Tokyo Women’s Medical University, Tokyo, Japan; Department of Cardiology, San Rossore Private Hospital and Medical Center, Pisa, Italy; Department of Cardiology, Heart Center Leipzig GmbH, Leipzig, Germany; Department of Cardiology, Na Homolce Hospital, Prague, Czech Republic; Department of Cardiovascular Medicine, Cleveland Clinic Foundation, Cleveland, OH, USA; Cardiac Rhythm Management, Abbott, Sunnyvale, CA, USA; Cardiac Rhythm Management, Abbott, Sunnyvale, CA, USA; Department of Cardiology and Electrophysiology, Amsterdam UMC, Amsterdam, The Netherlands

**Keywords:** Leadless pacemaker, Dual-chamber, Battery longevity, Aveir

## Abstract

**Aims:**

A dual-chamber leadless pacemaker (LP) system that employs distinct atrial and ventricular LP devices (ALP, VLP) has been introduced to clinical practice. Proprietary, low-energy, implant-to-implant (i2i) communication at each beat enables the devices to maintain synchronous atrioventricular sensing and pacing. We evaluated device longevities and contributing factors, such as i2i communication.

**Methods and results:**

Patients meeting dual-chamber pacing indications received the dual-chamber LP system as part of a prospective, multi-centre, international clinical trial (Aveir DR i2i Study, NCT05252702). Programming and diagnostics were interrogated from all *de novo*, non-revised, dual-chamber programmed devices at 12 months post-implant. This analysis included 302 patients (65% male; age 70 ± 13 years; weight 80 ± 19 kg; intrinsic heart rate 55 ± 7 bpm; 58% sinus node dysfunction, 27% atrioventricular block). At 12 months, devices were programmed to dual-chamber pacing (DDD(R) or DDI(R)) at a median 60 bpm rate, median 1.25 V pulse amplitude in ALP and 1.5 V in VLP, median 0.4 ms pulse width, and median i2i signal setting level 5 out of 7. Median ALP and VLP remaining battery longevities at 12 months were 4.3 and 9.1 years, with median total ALP and VLP longevities of 5.3 and 9.9 years. Base rate, pulse amplitude, pacing percentage, event rate, impedance, and i2i setting level all exhibited significant correlations with ALP and VLP longevities (*P* < 0.001). Programming i2i setting levels below 7 produced the greatest longevity savings.

**Conclusion:**

The first dual-chamber LP demonstrated adequate projected battery longevity after 12 months of use. Patient-specific device programming considerations, unique to leadless devices, may extend longevity.

## Graphical Abstract

**Graphical Abstract:**
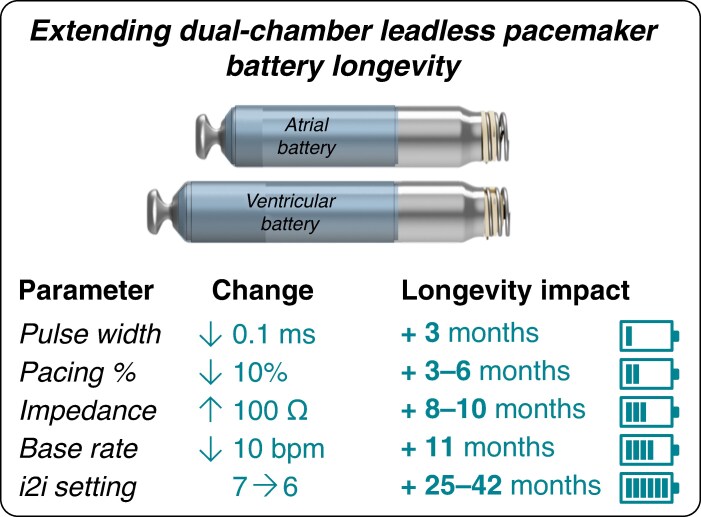

**What’s new?**

- A dual-chamber leadless pacemaker system has been released that includes distinct atrial and ventricular devices that communicate at each beat to maintain atrioventricular synchrony, but the battery longevity of this novel system has yet to be systematically studied.
- Battery longevities after 12 months of clinical use were evaluated as part of a prospective, multi-centre, international clinical trial.
- Median atrial and ventricular device longevities of 5.3 and 9.9 years were observed.
- Base rate, pulse amplitude, pacing percentage, event rate, impedance, and communication strength setting level all exhibited significant correlations with longevity.
- Patient-specific device programming considerations, unique to leadless devices, may extend longevity.

## Introduction

Leadless pacemakers (LPs) have eliminated the need for transvenous leads and a subcutaneous pulse generator pocket, thereby mitigating many of the associated complications.^1–4^ While the use of single-chamber, right ventricular (RV) LPs has been well established, the majority of pacemaker patients requires synchronous atrial and ventricular pacing. Recently, a modular, dual-chamber LP system has been developed that includes both a ventricular LP (VLP) and a smaller atrial LP (ALP) designed specifically to fit within the right atrium (RA). Such a leadless system is the result of decades of technological advances in cardiac pacing.^5,6^

Although the battery longevity of the VLP component of this pacemaker system has been previously reported,^7^ the battery longevity of the ALP component has not. Furthermore, the system employs implant-to-implant (i2i) communication between the two LPs at each atrial and ventricular event to maintain atrioventricular (AV) synchrony,^8,9^ which impacts the distinct battery longevities of each LP. A prospective, multi-centre, international, clinical trial of this technology has been conducted, with the safety, electrical performance, and resulting AV synchrony previously reported.^8,10,11^ The current report provides a focused evaluation of the projected battery longevities of both LP components of this dual-chamber leadless system after 12 months of use.

## Methods

### Device description

**Figure 1:**
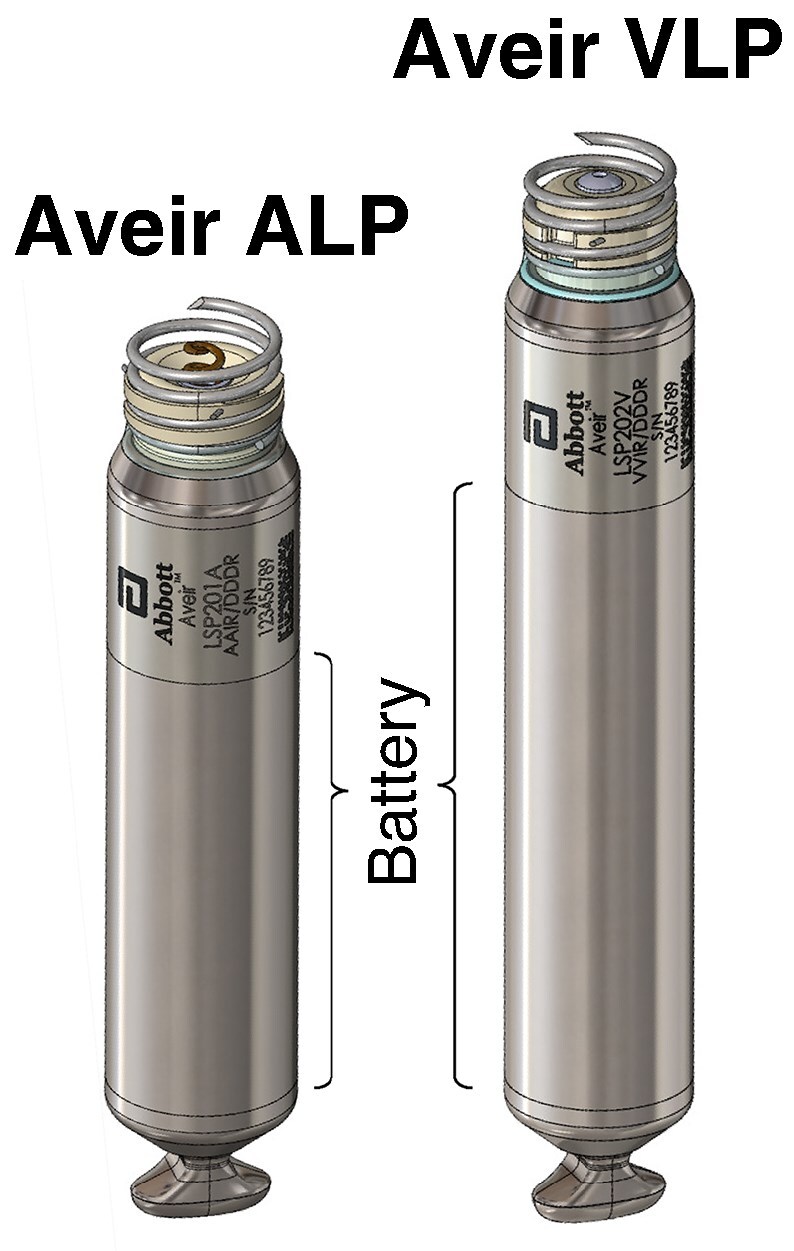
Schematics illustrating the Aveir DR leadless pacemaker system. Both the atrial and ventricular leadless pacemaker devices (ALP, VLP) are shown with the extents of their respective batteries.

The Aveir DR leadless pacemaker system (Abbott; Abbott Park, IL) has two completely independent but communicative devices: the ALP (length 32.2 mm, diameter 6.5 mm) and VLP (length 38.0 mm, diameter 6.5 mm), as shown in *Figure 1*. Although both LPs employ the same lithium-carbon monofluoride (Li-CFx) battery technology, the ALP is outfitted with a smaller sized battery than the VLP (174 vs. 241 mAh nominal capacity) to accommodate the shorter device length of the atrial device. Other ALP vs. VLP device differences have been described elsewhere.^11^

### Implant-to-implant communication

To maintain AV synchrony, implanted LPs communicate bidirectionally using the novel i2i communication protocol. Briefly, ALP-to-VLP and VLP-to-ALP transmissions in the form of subthreshold electrical pulses are sent immediately before each local paced event and after each local sensed event, allowing the receiving device to initiate the appropriate blanking periods, timers, and AV delay.^8,9,12^ The i2i signal characteristics can be tuned to enhance transmission success using the programmable i2i setting level, which incorporates the transmitting signal pulse amplitude, transmitting signal pulse duration, and receiving sensing threshold (range 1–7, nominal 4). Increasing the i2i setting level drains more current, particularly at the maximum setting of 7, which employs a significantly elevated i2i signal pulse amplitude.

### Clinical study design

Battery longevity was evaluated as part of a prospective, multi-centre, international, pivotal, clinical trial of the dual-chamber leadless pacing system (NCT05252702).^10^ Study inclusion criteria were the standard indications for dual-chamber pacing. Study exclusion criteria were mechanical tricuspid valve prosthesis, inferior vena cava filter, pre-existing pacing or defibrillation leads, or electrically-active implantable medical devices. The study was performed according to the principles outlined in the Declaration of Helsinki and the Good Clinical Practice guidelines of the European Commission. All patients provided written informed consent, and the study protocol was approved by each institutional ethics committee.

Following enrolment, the ALP/VLP system was implanted per standard instructions, with implant locations and device programming left to the discretion of the implanting physician. At 12 months post-implant (12M), each LP was interrogated using the Merlin programmer (Abbott, Abbott Park, IL), and the following values were collected: programmed settings (operating mode, pulse amplitude, pulse width, base rate, i2i setting level), pacing impedance, pacing percentage, and sensed/paced event rate. Note that the atrial and ventricular total event rates (i.e. total sensed and paced events/min) were used to quantify ALP-to-VLP and VLP-to-ALP i2i transmission rates, respectively, as a ventricular-based heart rate may not account for all atrial activity (e.g. automatic mode switch during atrial fibrillation or during AV block). Note that ALP-to-VLP i2i transmissions are sent for all atrial sensed events, even when automatic mode switch activates.

The estimated battery longevity remaining was calculated by the programmer using the battery capacity already consumed and the projected future consumption, based on the aforementioned parameters. Note that device diagnostics were cleared at 6 months (6M) post-implant to capture the most up-to-date 12M longevities. Consequently, all programmed and measured parameters may only reflect the 6M–12M post-implant window.

All study subjects with *de novo* implants (i.e. no existing pacing device already implanted), no subsequent device revision (e.g. post-implant LP replacements), dual-chamber pacing programmed (i.e. DDD(R) or DDI(R)), and complete longevity measurements at the 12M visit were included in this evaluation.

### Statistical analysis

Statistical analyses were performed using MATLAB (The Mathworks, Natick, MA). Demographic continuous variables are reported as mean ± standard deviation. All other continuous variables are reported as median [interquartile range (IQR)]. Correlations of continuous variables with battery longevity were quantified by Spearman’s rank correlation coefficients (ρ, range −1 to +1), and the associated longevity savings per unit change of each parameter was quantified by the slope of the line of best fit determined by linear regression.

Comparisons of proportions across two groups were evaluated using χ^2^ tests. Comparisons of continuous variables were evaluated using Mann–Whitney *U* tests across two groups or Kruskal–Wallis tests across 3+ groups, followed by *post hoc* Dunn’s tests with Sidak correction for multiple comparisons when warranted. In all tests, differences were considered statistically significant for *P* < 0.05. Battery longevities beyond the programmer display limit (i.e. ‘>25 years’) were conservatively considered 25.0 years for population statistics.

## Results

### Patient disposition and characteristics

From February 2022 to March 2023, 464 *de novo* patients were enrolled and 452 of 464 (97.4%) underwent an implant attempt across 77 centres in the USA, Canada, Europe, and Asia-Pacific. The 12 implants not attempted were due to either medical conditions precluding introducer sheath access or anti-coagulation, withdrawn consent, site implant allocations reached, or study inclusion criteria were no longer met. Of attempted implants, both LPs were successfully implanted in 446 of 452 (98.7%) subjects (four ALP implants were unsuccessful due to pericardial effusion, fixation, or exit block; two VLP implants were unsuccessful due to cardiac tamponade or fixation).

Among successfully implanted subjects, 24 of 446 subjects (5.4%) ultimately underwent device revision prior to 12M. The indications for revision included dislodgement of 11 ALP and 2 VLP; capture threshold elevation in two ALP, four VLP, and both in one patient; i2i communication issue in one patient; and upgrade to CRT-P due to indication changes in two patients. Of the remaining 422 subjects without device revision, 344 (81.5%) completed the 12M visit. Of those, 302 of 344 (87.8%) were programmed to a dual-chamber pacing mode (i.e. DDD(R) and DDI(R)), all of whom completed 12M ALP and VLP longevity data collection at 352 ± 25 days post-implant. Characteristics for those 302 subjects included in the evaluation are provided in *Table 1*.

**Table 1:** Baseline patient characteristics, device implant locations, and programmed pacing mode for all evaluated subjects

Characteristic	Value
*N*	302
Male birth sex, *n* (% patients)	197 (65.2%)
Age, years	70.3 ± 13.3
Weight, kg	80.0 ± 18.8
Height, cm	170.4 ± 11.2
Body surface area, m^2^	1.9 ± 0.3
Ejection fraction, %	59.8 ± 6.6
History of atrial fibrillation, *n* (% patients)	101 (33.4%)
Intrinsic heart rate, bpm	55.4 ± 7.0
Indication, % patients	
Sinus node dysfunction	57.9
AV block, 1st degree	4.6
AV block, 2nd degree	6.3
AV block, 3rd degree	15.6
Other	15.6
ALP RA implant location, % patients	
Anterior RAA base	47.4
Posterior RAA base	14.9
Mid-to-deep RAA	21.8
RA lateral wall	9.3
Other	6.6
VLP RV implant location, % patients	
Apical septum	51.7
Mid septum	36.8
Apex	7.3
Other	4.2

Values are shown as count with per cent, or as mean ± standard deviation where appropriate.

### Dual-chamber battery longevity

**Figure 2:**
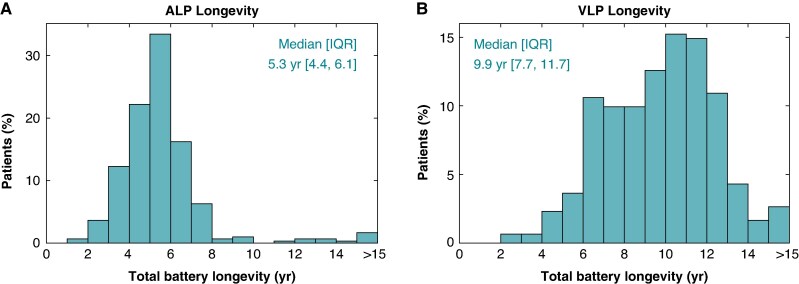
Leadless pacemaker device battery longevities. Total battery longevities observed for (*A*) ALP and (*B*) VLP devices, as projected from measurements at 12M.

Distributions of the ALP and VLP total battery longevity estimates are provided in *Figure 2*. Median [IQR] remaining ALP and VLP longevities were 4.3 years [3.5, 5.1] and 9.1 years [6.8, 10.7] after 12 months of use. Including the elapsed time post-implantation, the median total ALP and VLP longevities were 5.3 years [4.4, 6.1] and 9.9 years [7.7, 11.7], respectively.

### Programmed parameters

**Figure 3:**
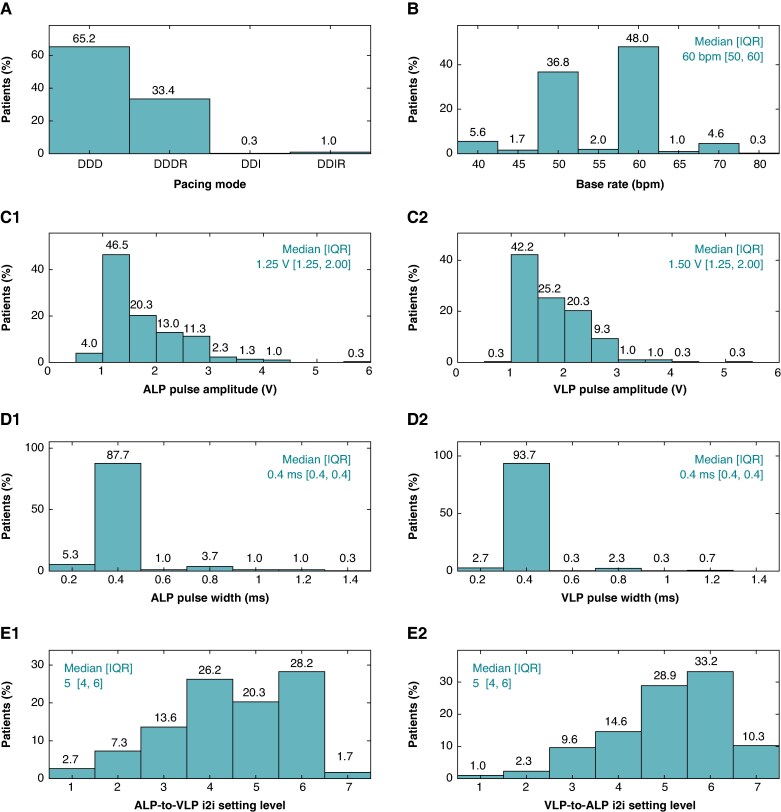
Programmed parameters distributions at 12 months. (*A*) Pacing mode. (*B*) Base rate. (*C*) ALP and VLP pulse amplitudes. (*D*) ALP and VLP pulse widths. (*E*) ALP and VLP i2i setting levels.

Programmable parameters expected to significantly impact battery longevity include pacing mode, rate-response, base rate, pulse amplitude, pulse width, and i2i setting levels. The distributions of these parameters programmed at the start of the 12M visit are provided in *Figure 3*. LP systems were predominantly programmed to DDD (65.2%) or DDDR (33.4%), as shown in *Figure 3A*. The median programmed base pacing rate was 60 bpm [50, 60] (*Figure 3B*). Atrial leadless pacemakers were programmed at a median pulse amplitude of 1.25 V [1.25, 2.0], with 87.7% at 0.4 ms pulse width; VLPs were programmed at a median pulse amplitude of 1.5 V [1.25, 2.0], with 93.7% at 0.4 ms pulse width (*Figure* *3C* and *D*). Median programmed ALP-to-VLP and VLP-to-ALP i2i setting levels were both 5 [4, 6] out of 7 (*Figure 3E*).

### Measured parameters

**Figure 4:**
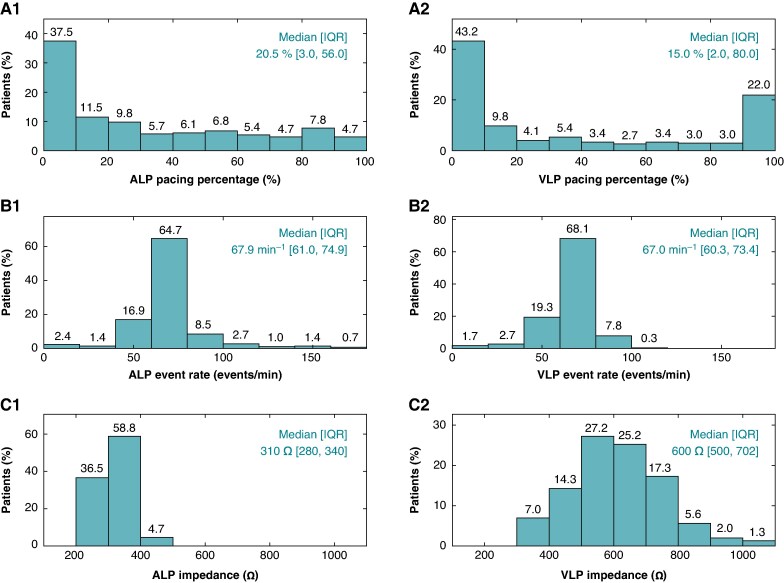
Distributions of device measurements interrogated at 12 months. (*A*) ALP and VLP lifetime pacing percentages. (*B*) ALP and VLP lifetime paced and sensed event rates. (*C*) ALP and VLP pacing impedances.

Device diagnostic measurements expected to correlate with battery longevity include pacing percentage (i.e. due to delivery of pacing impulses), event rate (i.e. total sensed and paced events, due to the transmission of i2i messages whenever atrial or ventricular activity occurs), and impedance (i.e. impacting the current required to achieve the programmed pulse amplitude). The distributions of these measurements interrogated at the start of the 12M visit are provided in *Figure 4*. Median ALP and VLP pacing percentages were 20.5% [3.0, 56.0] and 15.0% [2.0, 80.0], respectively (*Figure 4A*). Atrial leadless pacemaker pacing was delivered in <10% of cycles in 37.5% of subjects; VLP pacing was delivered in <10% of cycles in 43.2% of subjects. Atrial leadless pacemaker and VLP pacing or sensing events occurred at median rates of 67.9 events/min [61.0, 74.9] and 67.0 events/min [60.3, 73.4], respectively (*Figure 4B*). Median ALP and VLP pacing impedances of 310 Ω [280, 340] and 600 Ω [500, 702] were observed, respectively (*Figure 4C*).

### Battery longevity extremes

Focusing on LPs with longevities in the top and bottom 10th percentiles may help explain contributing factors. Notable differences in ALPs with remaining longevities in the top 10th percentile (>7.2 years, *N* = 30) included a lower programmed base rate, pulse amplitude, ALP-to-VLP i2i setting level, atrial pacing percentage, and atrial event rate than the rest of the population (*P* < 0.05 for each). Ventricular leadless pacemakers with remaining longevities in the top 10th percentile (>12.9 years, *N* = 31) exhibited a lower programmed base rate, VLP-to-ALP i2i setting level, ventricular pacing percentage, and ventricular event rate than the rest of the population, but exhibited a higher impedance and included more female patients (*P* < 0.05 for each).

Atrial leadless pacemakers with remaining longevities in the bottom 10th percentile (<3.3 years, *N* = 28) were associated with a higher programmed base rate, pulse amplitude, pulse width, and ALP-to-VLP i2i setting level than the rest of the population, exhibited a lower impedance, and included more patients with rate-response enabled and/or a history of atrial fibrillation (*P* < 0.05 for each). Similarly, VLPs with remaining longevities in the bottom 10th percentile (<6.2 years, *N* = 30) were associated with a higher programmed base rate, pulse amplitude, pulse width, VLP-to-ALP i2i setting level, pacing percentage, height, weight, and body surface area than the rest of the population, but exhibited a lower impedance and included more male patients (*P* < 0.05 for each).

### Correlations with battery longevity

The distinct impact of each programmed or measured parameter on battery longevity were quantified using Spearman’s rank correlation coefficients and linear slopes, as provided in *Table 2*. All of the aforementioned parameters that were expected to impact battery longevity (i.e. base rate, pulse amplitude, pulse width, i2i setting level, pacing percentage, event rate, and impedance) exhibited statistically significant correlations with ALP battery longevity (*P* < 0.001 for each). Results were similar for VLP battery longevity, although the impact of pulse width failed to achieve statistical significance. In both devices, correlation coefficients were limited to approximately ±0.5 out of a maximum of ±1.0.

**Table 2:** Parameter correlations with battery longevity^a^

Parameter	Correlation coefficient (*P*-value)	Estimated longevity impact
ALP		
Base rate	−0.39 (*P* < 0.001)	+11.2 months per 10 bpm decrease
Pulse amplitude	−0.36 (*P* < 0.001)	+10.8 months per 1.0 V decrease
Pulse width	−0.23 (*P* < 0.001)	+3.1 months per 0.1 ms decrease
Pacing percentage	−0.35 (*P* < 0.001)	+2.6 months per 10% decrease
Event rate (sensed + paced)	−0.30 (*P* < 0.001)	+6.3 months per 10 events/min decrease
Impedance	+0.30 (*P* < 0.001)	+10.5 months per 100 Ω increase
ALP-to-VLP i2i setting level	−0.32 (*P* < 0.001)	+3.1 months per unit decrease ≤6 + 25.2 months when decreased from 7
Rate-response	N/A	+10.3 months when disabled
VLP		
Base rate	−0.22 (*P* < 0.001)	+11.7 months per 10 bpm decrease
Pulse amplitude	−0.23 (*P* < 0.001)	+11.2 months per 1.0 V decrease
Pulse width	−0.11 (*P* = 0.07)	Not significant
Pacing percentage	−0.52 (*P* < 0.001)	+4.0 months per 10% decrease
Event rate (sensed + paced)	−0.23 (*P* < 0.001)	+5.4 months per 10 events/min decrease
Impedance	+0.35 (*P* < 0.001)	+8.2 months per 100 Ω increase
VLP-to-ALP i2i setting level	−0.37 (*P* < 0.001)	+3.7 months per unit decrease ≤6 + 41.9 months when decreased from 7
Rate-response	N/A	+2.5 months when disabled

^a^Spearman’s rank correlation coefficients are provided for continuous parameters, along with linear fit estimations of longevity impact resulting from changes in parameter values.

Lines of best fit were calculated to estimate the impact of parameter changes on longevity. For ALP and VLP devices, a longevity savings of ∼11 months resulted from each 10 bpm decrease in programmed base rate or each 1.0 V decrease in pulse amplitude. Atrial leadless pacemaker longevities were extended by 3 months per 0.1 ms decrease in pulse width. For ALP and VLP devices, each decrease in pacing percentage by 10% or event rate by 10 events/min extended longevity by ∼3–6 months. Atrial leadless pacemaker and VLP impedance showed the only positive correlation, with each 100 Ω increase extending longevity by 8–10 months.

Piece-wise linear fits were used to describe the impact of i2i setting level, due to the non-linear impact of elevating the i2i setting level from 6 to 7. For ALP and VLP devices, each step down in the programmed i2i setting level below 6 exhibited a longevity savings of 3–4 months. In contrast, decreasing the i2i setting level from 7 to 6 extended longevity by ∼2 years in ALPs (i.e. for ALP-to-VLP i2i setting level) and ∼3.5 years in VLPs (i.e. for VLP-to-ALP i2i setting level).

The 198 (65.6%) subjects in whom rate-response was disabled (i.e. DDD or DDI), when compared to the 104 (34.4%) subjects with rate-response enabled (i.e. DDDR or DDIR), exhibited significantly longer ALP longevities (5.6 [4.9, 6.4] vs. 4.8 years [3.8, 5.5]; *P* < 0.001), but similar VLP longevities (10.0 years [7.7, 11.7] vs. 9.8 years [7.8, 11.5]; *P* = 0.96). The impact of disabling atrial tracking (i.e. DDI(R)) could not be evaluated, as only four (1.3%) subjects had such programming.

The indication for pacing, which defines the programmed settings used for treatment, had an impact on longevity. Atrial leadless pacemaker longevity was shorter in the 175 (57.9%) sinus node dysfunction patients vs. all others (5.0 [4.1, 5.9] vs. 5.6 [5.0, 6.4], *P* < 0.001), concordant with a higher programmed base rate, higher atrial pacing percentage, and greater proportion with rate-response enabled (*P* < 0.001 for each). In contrast, VLP longevity was longer in sinus node dysfunction patients (10.6 [8.6, 12.2] vs. 8.9 [7.1, 10.8], *P* < 0.001), concordant with a lower ventricular pulse amplitude and ventricular pacing percentage (*P* < 0.05 for each). As expected, VLP longevities were shorter for patients with 3rd degree heart block and at least 90% ventricular pacing (7.9 [6.7, 9.0] vs. 10.2 [8.0, 11.8], *P* < 0.005).

**Figure 5:**
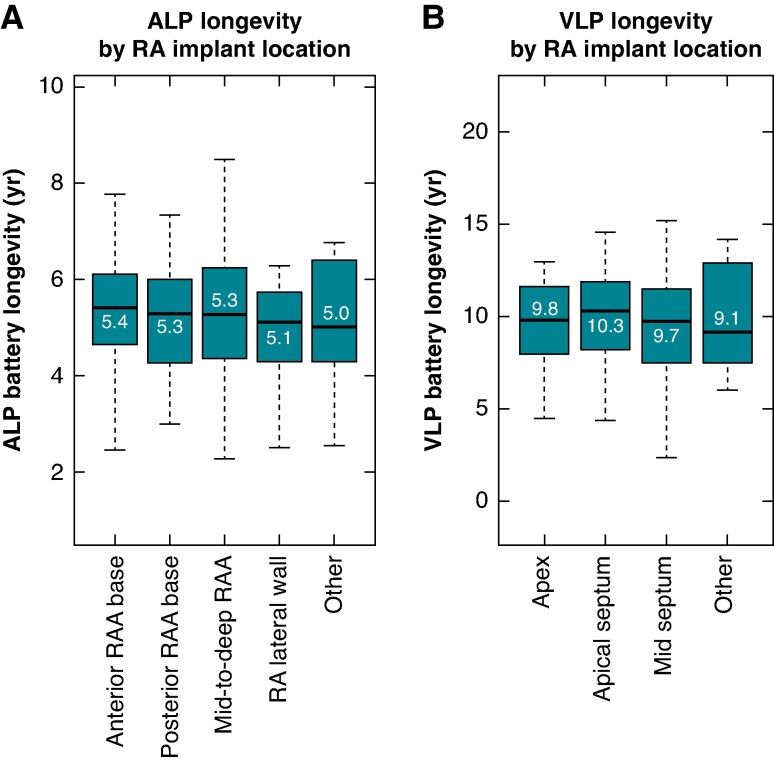
Battery longevities according to RA and RV implant location. (*A*) ALP longevities grouped by RA implant location. (*B*) VLP longevities grouped by RV implant location. Neither ALP (*P* = 0.81) nor VLP (*P* = 0.35) demonstrated significant differences in battery longevity across implant location.

When comparing battery longevities across RA or RV implant locations, neither the ALP (*P* = 0.81) nor VLP (*P* = 0.35) demonstrated significant group-wise differences, as shown in *Figure 5* (see *Table 1* for implant location distributions).

## Discussion

This report provides an in-depth, *post hoc* evaluation of the projected battery longevities of the atrial and ventricular leadless pacemakers that comprise the dual-chamber LP system. In the 302 *de novo* dual-chamber LP systems successfully implanted without subsequent device revision and programmed to a dual-chamber pacing mode, median total ALP and VLP longevities of 5.3 and 9.9 years were observed, respectively.

Devices in this population demonstrated a broad range of battery longevities resulting from variability in both programming and pacing demand. Atrial leadless pacemaker and VLP devices with longevities in the top 10th percentile were associated with a lower programmed base rate, i2i setting level, pacing percentage, and event rate. Both ALP and VLP devices with longevities in the bottom 10th percentile were associated with a higher programmed base rate, pulse amplitude, pulse width, and i2i setting level, but a lower impedance. These factors, with the exception of event rate and impedance, can be influenced by device reprogramming, which should be considered on a patient-by-patient basis to boost longevity. Atrial leadless pacemaker devices with longevities in the bottom 10th percentile also included more patients with a history of atrial fibrillation, as bouts of rapid atrial activity (i.e. atrial events, even during automatic mode switch) trigger more ALP-to-VLP i2i transmissions; the impact of atrial fibrillation on VLP longevity may have been curbed by limited AV conduction (fewer ventricular sensed events) and/or limited atrial tracking (fewer ventricular paced events when automatic mode switch was enabled), resulting in fewer VLP-to-ALP i2i transmissions.

In the broader study population, several programmable parameters and patient-specific device measurements demonstrated statistical correlations with battery longevity. As expected, base rate, pulse amplitude, pulse width, pacing percentage, event rate, and i2i setting level were all negatively correlated with the longevity of their respective LP, with the exception of pulse width for the VLP; in contrast, impedance was positively correlated with longevity. Factors that are consistent with longevity trends in transvenous pacemakers include (i) pulse amplitude and pulse width (both influenced by impedance), which determine the current consumed for each pacing stimulus, and (ii) pacing percentage (influenced by the base rate) and event rate, which determine the frequency of pacing stimuli delivered.^13^ Unique to this dual-chamber leadless pacing system is the current consumed by i2i communication, which is impacted by the event rate (total sensed and paced), impedance, and i2i setting level.

In terms of the relative impact of each parameter on projected longevity, programming the i2i setting level below the maximum (i.e. 7) was associated with a particularly notable battery savings due to the number of transmissions sent in each direction, for paced and sensed events alike. This population exhibited ∼70 distinct atrial and ventricular events/min, resulting in ∼70 ALP-to-VLP and 70 VLP-to-ALP transmissions every minute (i.e. one transmission for each atrial or ventricular sensed activity or paced impulse). Devices set to the maximum i2i setting level send each of these i2i transmissions with a particularly high signal amplitude.^9^ If longevity is of concern for a specific patient, i2i setting levels should be titrated down, as is commonly done with pulse amplitudes, until the requisite i2i communication success for that patient is achieved.

For example, a patient with isolated sinus node dysfunction (i.e. with intact AV conduction) may not require continuous atrial tracking, so ALP-to-VLP i2i transmissions may not be prioritized, and the ALP-to-VLP i2i setting level may be reduced. In contrast, a patient with AV block but an intact sinus node may not require atrial pacing, so VLP-to-ALP i2i transmissions may not be prioritized, and the VLP-to-ALP i2i setting level may be reduced. Avoiding the maximum setting of 7, in particular, extended the ALP longevity by 2 years and VLP longevity by 3.5 years in the entire study population.

Interestingly, these i2i-related battery savings may counter the increased consumption associated with pacing demand for specific patient populations. For example, sinus node dysfunction patients were associated with a shorter ALP longevity due, in part, to an elevated atrial pacing percentage. However, ALP-to-VLP i2i setting levels may be reduced to counter this consumption if atrial tracking is not needed. Similarly, AV block patients were associated with shorter VLP longevity due, in part, to an elevated ventricular pacing percentage. To counter this consumption, VLP-to-ALP i2i settings in these patients may be reduced if atrial pacing is not needed (i.e. healthy sinus node function). Note that longevity-extending programming considerations associated with traditional transvenous pacing systems still apply for this leadless system; examples include (i) programming AAI(R), reducing the base rate, enabling rate hysteresis, or reducing rate-response sensitivity for sinus node dysfunction patients with intact AV conduction, or (ii) programming VVI(R) for patients with intermittent AV block but intact sinus node function.

Regardless of pacing indication and prospective i2i-related battery consumption, implant procedure considerations can also play an important role. Implanting the two LPs with minimal distance between them and minimal relative angle (i.e. aligned) can enhance communication and reduce the i2i setting levels needed to maintain successful communication.^9^ The absence of significant longevity differences across RA or RV implant locations in this study may be attributed to: (i) differences in heart size and chamber anatomy among patients that may vary the ALP–VLP angle and distance, and thus i2i communication success and requisite i2i setting level, associated with a particular implant location^8^; (ii) insufficient adjustment of i2i setting levels in response to measured i2i success; or (iii) confounding population-wide differences in programming, impedance, or pacing demand.

The total VLP longevities observed in this study with rate-response either enabled or disabled (9.8 years enabled, 10.0 years disabled) were comparable to previous manufacturer-agnostic reports of 2000+ traditional transvenous dual-chamber pacemakers (roughly 7 years enabled, 9 years disabled).^14^ Although transvenous dual-chamber pulse generators deliver both atrial and ventricular pacing/sensing, the leadless system would be limited by the relatively shorter total ALP longevity (4.8 years rate-response enabled, 5.6 years disabled). While the aforementioned implant location and programming mitigations may extend ALP longevity, the modular nature of this leadless system allows replacement of just the ALP as needed.

Although a dual-chamber leadless pacing alternative is not currently available for longevity comparisons, a leadless VDD-capable system (Micra AV; Medtronic Inc., Minneapolis, MN) demonstrated a median total longevity of 10.5 years for commercial devices. Aside from population-level differences in pacing demand, the roughly 6-month longevity difference between that VDD device and the DDD VLP device in the current report can be attributed to the battery consumption associated with i2i transmissions at each atrial and ventricular event. Specifically, the VLP-specific longevity in DDD mode reflects (i) transmitting VLP-to-ALP i2i signals to time atrial pacing and (ii) sensing ALP-to-VLP i2i signals to track atrial activity, both of which are needed to maintain AV synchrony. Some VDD-indicated patients, however, may benefit from the atrial pacing delivered by a DDD-capable device, despite the slight longevity cost. It may also be possible for ALP implants to supplement patients with existing VDD devices who later require atrial pacing, effectively providing a form of DDD functionality without i2i communication.

While adequate battery longevities were demonstrated by this dual-chamber leadless system after 12 months of use, patient-specific programming considerations, including progressive monitoring for optimization opportunities, may further extend these longevities. Pulse amplitude and/or pulse width safety margins, for example, should be re-evaluated over time and minimized. In addition, avoiding the maximum i2i setting level and reducing the base pacing rate, when possible, will reduce the amplitude and frequency of i2i transmissions, consequently prolonging battery life.

As this prospective trial captured the first clinical experience with this novel pacing system, some parameters critical to battery consumption (e.g. pulse amplitude, pulse width, i2i setting level, and PCT safety margin) may have been programmed more conservatively by physicians as a precaution. As experience with this novel pacing system and its programming optimization accrues, even greater real-world battery longevities may be expected. Furthermore, a new programming option has been introduced that delivers atrial pacing with backup ventricular pacing when needed (AAI + VVI backup), intended for patients with largely intact atrioventricular conduction and limited ventricular pacing needs. In the future, advancements in battery design and improved consumption profiles can be expected that will provide extended longevity, which would be particularly valuable in volume-limited atrial devices.

### Limitations

Device programming was left to the discretion of the clinician and could have been altered over the course of the study. Consequently, the pacing mode and other programmed parameters interrogated at 12M only reflect the programming since the previous in-clinic visit (i.e. 6M). Alternative programming in the first 6 months of use may have influenced 12M longevities. However, as higher pulse amplitudes, pulse widths, and i2i setting levels may have been programmed in the first few months of implant as a precaution in the first clinical use of this novel device, the 12M longevities reported here were likely underestimated, rather than overestimated.

Longevity savings values for each parameter were based on population-wide lines of best fit, without controlling for other confounding variables. However, the population included a broad range of all measured and programmed variables, which may wash out any inter-variable associations.

## Conclusions

This dual-chamber LP system demonstrated adequate battery longevity after 12 months of use. Patient-specific implant location and device programming considerations, unique to leadless devices, may extend longevity.

## Data Availability

The data underlying this article will be shared on reasonable request to the corresponding author.
